# Platelet-derived β2m regulates age related monocyte/macrophage functions

**DOI:** 10.18632/aging.102520

**Published:** 2019-12-18

**Authors:** Zachary T. Hilt, Sara K. Ture, Amy Mohan, Allison Arne, Craig N. Morrell

**Affiliations:** 1 Aab Cardiovascular Research Institute, University of Rochester School of Medicine, Box CVRI, Rochester, NY 14652, USA; 2 Department of Microbiology and Immunology, University of Rochester School of Medicine, Rochester, NY 14652, USA

**Keywords:** platelets, monocytes, inflammation, aging, heart

## Abstract

Platelets have central roles in both immune responses and development. Stimulated platelets express leukocyte adhesion molecules and release numerous immune modulatory factors that recruit and activate leukocytes, both at the sites of activation and distantly. Monocytes are innate immune cells with dynamic immune modulatory functions that change during the aging process, a phenomenon termed “inflammaging”. We have previously shown that platelets are a major source of plasma beta-2 microglobulin (β2M) and that β2M induced a monocyte pro-inflammatory phenotype. Plasma β2M increases with age and is a pro-aging factor. We hypothesized that platelet derived β2M regulates monocyte phenotypes in the context of aging. Using wild-type (WT) and platelet specific β2M knockout mice (Plt-β2M^-/-^) mice, we found that plasma β2M increased with age and correlated with increased circulating Ly6C^Hi^ monocytes. However, aged Plt-β2M^-/-^ mice had significantly fewer Ly6C^Hi^ monocytes compared to WT mice. Quantitative real-time PCR of circulating monocytes showed that WT mouse monocytes were more “pro-inflammatory” with age, while Plt-β2M^-/-^ derived monocytes adopted a “pro-reparative” phenotype. Older Plt-β2M^-/-^ mice had a significant decline in heart function compared to age matched WT mice, as well as increased cardiac fibrosis and pro-fibrotic markers. These data suggest that platelet-derived β2M regulates age associated monocyte polarization, and a loss of platelet derived β2M shifted monocytes and macrophages to a pro-reparative phenotype and increased pro-fibrotic cardiac responses. Platelet regulation of monocyte phenotypes via β2M may maintain a balance between inflammatory and reparative signals that affects age related physiologic outcomes.

## INTRODUCTION

Platelets are best known for their role in thrombosis and hemostasis. However, they have central roles in regulating all facets of immune responses and are immune regulatory cells [1]. Leukocytes can be regulated by platelets through contact dependent (PSGL-1/CD62P, Mac-1/GPIbα) [2] and contact independent mechanisms (release of granule contents and *de novo* mediator production) [1]. Platelets express major histocompatibility complex I (MHC I), and they have the potential to present antigens [3]. Beta-2 microglobulin (β2M) is a chaperone molecule for MHC I cell surface trafficking and stability [4]. β2M is neither a transmembrane protein, nor covalently bound to the MHCα chain, making β2M easily shed into the plasma [4]. Elevated plasma β2M is associated with increased risk for multiple inflammatory processes, including cardiovascular disease (CVD) [5] and age related neurocognitive decline [6]. Platelets express abundant β2M, and β2M is in the activated platelet releasate [7]. Using platelet specific β2M^-/-^ mice (Plt-β2M^-/-^) generated by our lab, we previously reported that platelets are the major source of plasma β2M and that platelet derived β2M has direct pro-inflammatory effects on monocytes, independent of MHC I trafficking functions [8].

Aging is the greatest risk factor for cardiovascular disease (CVD) and the leading cause of death in those 65 and older. By 2030, 20% of the population will be over 65, increasing the impact of age associated CVD on the healthcare system [9]. As the heart ages, there is an increase in cell apoptosis, senescence, ischemic tissue damage, and fibrosis [9]. Macrophages originate from two major sources in post-neonatal development: yolk sac derived that make up tissue resident populations (Kupffer, Langerhans, microglia) [10] and monocyte-derived populations [11] that are recruited during pathological tissue inflammation or into tissue that has low-grade homeostatic inflammation, such as the intestine. Yolk sac derived tissue resident cardiac macrophages initially proliferate, but with age, circulating monocytes replace yolk sac derived macrophages [12]. CVD, such as myocardial infarction (MI), results in recruited monocyte subsets that have critical roles in regulating heart injury repair and changes in heart function [13, 14]. Human monocytes are divided into three subsets: 1) classical monocytes (CD14^++^ CD16^-^) that are phagocytic and release reactive oxygen species and pro-reparative cytokines (IL-10); 2) intermediate monocytes (CD14^++^ CD16^+^) characterized by a pro-inflammatory cytokine profile (TNFα, IL-1β); 3) non-classical monocytes (CD14^+^ CD16^++^) that have patrolling characteristics and a pro-inflammatory cytokine profile [15, 16]. Mice are typically described as having two circulating monocyte subsets: 1) Ly6C^Hi^ monocytes that are pro-inflammatory and phagocytic; 2) Ly6C^Lo^ monocytes that are pro-reparative, pro-fibrotic, and exhibit a patrolling behavior [15, 16].

Macrophage polarization is most commonly characterized into two subtypes: M1 and M2 [17]. The nomenclature of M1/M2 were originally defined through *in vitro* experiments using various agonist that differentially polarized macrophages; but because *in vivo* macrophages likely exist on a spectrum, have multiple possible agonists, and exhibit plasticity [18] we call macrophages “M1-like” and “M2-like” for simplicity. M1-like macrophages are characterized *in vitro* by stimulation using LPS, IFN-γ, GM-CSF, phenotypically characterized by high surface expression of MHC II and inducible nitric oxide synthase (iNOS) [15, 17]. Functionally, M1-like macrophages are professional killers that participate in phagocytosis of cellular debris, promote proteolysis and the turnover of extracellular matrix, present antigens to lymphocytes and release inflammatory cytokines [12, 15]. M2-like macrophages are stimulated *in vitro* by IL-4, IL-13, IL-10, TGFβ1, M-CSF, and are phenotypically characterized by CD206 surface expression and production of arginase1 (Arg1) [15, 17]. M2-macrophages functionally promote angiogenesis, wound healing, tissue fibrosis, ECM production and the release anti-inflammatory cytokines [12, 15].

Aging leads to changes to the immune system. “Inflammaging” is a term used to characterize chronic, low-grade, inflammation that occurs in the elderly and includes changes to post-translationally modified proteins, increased cell senescence, and altered plasma concentrations of inflammatory cytokines [19, 20]. Previous reports have characterized monocyte inflammaging phenotypically and functionally as a pro-inflammatory phenotype (non-classical CD16^Hi^ in humans, Ly6C^Hi^ in mice) [21–23]. Based on these data, we hypothesized that the recruitment of circulating monocytes into the heart during the aging process has a role in regulating heart function and that platelet derived β2M’s polarization of monocytes may be a central regulator of these responses.

## RESULTS

### During the aging process, increased plasma β2M correlates with increased circulating pro-inflammatory monocytes

**Figure 1:**
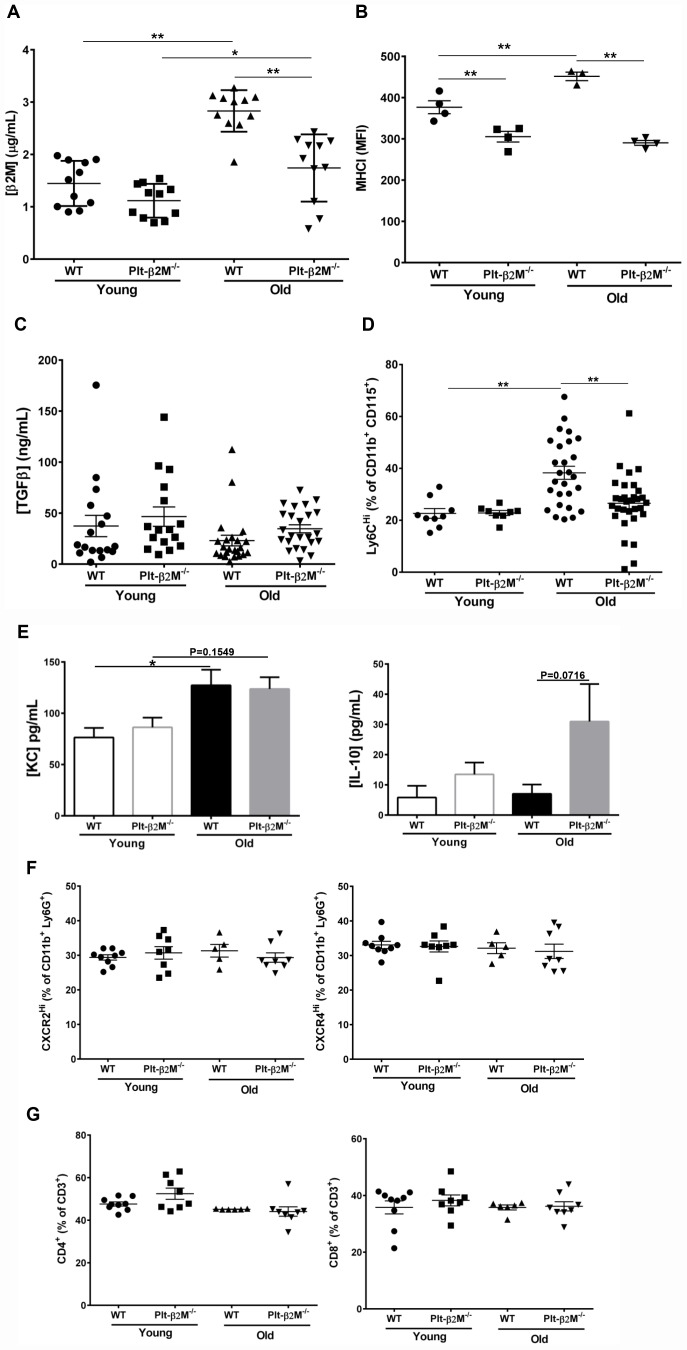
**A lack of platelet-derived β2M changes age associated monocyte phenotypes.** (**A**) Platelets are a major source of age associated increases in plasma β2M. Plasma was isolated from 2-4 and 14-16 mos old WT and Plt-β2M^-/-^ mice and β2M was quantified by ELISA. Plasma β2M had a greater increase with age in WT compared to Plt-β2M^-/-^ mice (N=11, mean ± SEM, *P<0.05, **P<0.01, one-way ANOVA with Bonferroni correction). (**B**) Platelet MHC I increased with age. Platelets were isolated from 4 and 14 mos old WT and Plt-β2M^-/-^ mice. Surface MHC I was quantified by flow cytometry. MHC I increased with age in WT, but not Plt-β2M^-/-^ mice (N=3-4, mean ± SEM, **P<0.01, one-way ANOVA with Bonferroni correction). (**C**) Plasma TGFβ does not significantly change with age in either WT or Plt-β2M^-/-^ mice. Plasma TGFβ was quantified by ELISA (N=17-24, mean ± SEM, one-way ANOVA with Bonferroni correction). (**D**) Aged Plt-β2M^-/-^ mice had fewer circulating Ly6C^Hi^ monocytes compared to WT mice. Peripheral blood was isolated from 4 mos and 14 mos old WT and Plt-β2M^-/-^ mice and monocyte Ly6C expression determined by flow cytometry. Ly6C^Hi^ monocytes were increased in older WT, but not Plt-β2M^-/-^ mice (N=8-31, mean ± SEM, **P<0.01, one-way ANOVA with Bonferroni correction). (**E**) 14 mos old WT mice have increased plasma KC while Plt-β2M^-/-^ have increased plasma IL-10 (N=16-22, mean ± SEM, one-way ANOVA with Bonferroni correction). (**F**) 14 mos old WT and Plt-β2M^-/-^ mice had similar circulating neutrophils. Peripheral blood was isolated from 4 mos and 14 mos old WT and Plt-β2M^-/-^ mice and CXCR2^Hi^ and CXCR4^Hi^ neutrophils were quantified by flow cytometry (N=5-9, mean ± SEM, one-way ANOVA with Bonferroni correction). (**G**) 14 mos old WT and Plt-β2M^-/-^ mice have similar numbers of circulating T cells. Peripheral blood was isolated from 4 mos and 14 mos old WT and Plt-β2M^-/-^ mice and CD4 and CD8 T cells quantified by flow cytometry (N=6-9, mean ± SEM, one-way ANOVA with Bonferroni correction).

We previously demonstrated that platelets are a major source of circulating plasma β2M. PF4^Cre^-β2M^Flox/Flox^ (Plt-β2M^-/-^) mice specifically lack β2M in platelets and had reduced plasma β2M compared to wild-type (WT) control mice [8]. Plasma β2M increases with age in both mice and humans [6]. Old (> 14 months) WT mice housed in standard conditions had a significant increase in plasma β2M compared to young (< 4 months) WT mice (Figure 1A). Old Plt-β2M^-/-^ mice had an increase in plasma β2M compared to young genotype controls, however the plasma levels were still significantly lower than old WT mice (Figure 1A). There was a significant increase in age associated platelet counts in both WT and Plt-β2M^-/-^ mice (Table 1). This suggests that platelets may be a major source of an age-dependent increase in plasma β2M. Platelets from old WT mice had increased surface MHC I compared to young genotype control mice, while Plt-β2M^-/-^ mice did not (Figure 1B). Compared to young mice, there was a significant increase in P-selectin surface expression on old WT and old Plt-β2M^-/-^ isolated platelets, both without agonist stimulation and in a dose dependent manner in response to ADP and high thrombin concentrations (Supplementary Figure 1). β2M and TGFβ can act antagonistic to each other [8, 24], thus we measured plasma TGFβ. There was no significant difference in plasma TGFβ between young or old mice in either genotype (Figure 1C), while there was a insignificant trend in increased plasma TGFβ in Plt-β2M^-/-^ mice compared to WT mice (Figure 1C). These results are similar to human data that found either no difference, or a decrease, in plasma TGFβ with age [25, 26].

**Table 1:** WT and Plt-β2M^-/-^ complete blood counts.

	**WT 4 mos (n=5)**	**WT 14 mos (n=22)**	**Plt-β2M^-/-^4 mos (n=7)**	**Plt-β2M^-/-^ 14 mos (n=26)**
WBC	12.6 ± 3	15.4 ± 3.97	12.4 ± 2.5	13.1 ± 2.67
Lymphocytes	9.8 ± 3.5	12.9 ± 3.23	10.3 ± 1.7	11.0 ± 2.30
Monocytes	0.46 ± 0.2	0.32 ± 0.17	0.31 ± 0.14	0.28 ± 0.19
Neutrophils	2.4 ± 0.6	2.00 ± 0.93	2.0 ± 0.7	1.88 ± 0.80
RBC	10.6 ± 0.5	9.99 ± 0.68	10.6 ± 0.3	10.0 ± 0.69
Platelets	524 ± 98	780 ± 155	592 ± 98	950 ± 148**

Because both β2M and TGFβ are circulating factors that can directly polarize monocytes [8, 27, 28] we characterized circulating monocyte subsets. WT and Plt-β2M^-/-^ mice had no difference in the total number of circulating monocytes (Table 1), but there was a significant increase in the percentage of Ly6C^Hi^ monocytes in old WT mice compared to both WT young and Plt-β2M^-/-^ old mice (Figure 1D). Ly6C^Hi^ monocytes produce pro-inflammatory cytokines such as KC (CXCL1), while Ly6C^Lo^ monocytes produce pro-reparative cytokines such as (IL-10). Because of the difference in the percentage of Ly6C^Hi^ monocytes during aging and in presence of platelet derived β2M, we determined whether plasma levels of either were changed. Old WT mice had a significant increase in plasma KC, while old Plt-β2M^-/-^ mice had a trending, but not significant, increase in KC compared to young genotype controls (Figure 1E). Conversely, old Plt-β2M^-/-^ mice had an increase in plasma IL-10 compared to young genotype controls, while WT mice had no age associated change in plasma IL-10 (Figure 1E). There was no difference in total neutrophil count (Table 1) and flow cytometry analysis of circulating neutrophils showed no phenotypic difference between WT and Plt-β2M^-/-^, whether young or old (Figure 1F). We also determined the number of circulating lymphocytes (Table 1), total CD3^+^ T cells (Supplementary Figure 2), and the percentage of CD4^+^ and CD8^+^ T cells (Figure 1G). There were no differences between any age or mouse genotype in these cell types.

### Circulating monocytes are phenotypically and functionally different during aging

**Figure 2:**
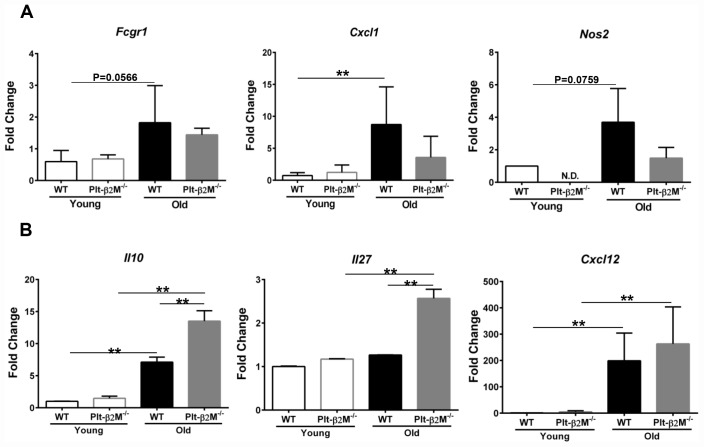
**Monocytes from Plt-β2M^-/-^ mice had a more reparative gene expression pattern.** (**A**) Circulating monocytes from aged WT, but not Plt-β2M^-/-^ mice, had increased inflammatory gene expression. Peripheral blood monocytes were isolated and qRT-PCR for inflammatory associated gene markers, *Fcgr1*, *Cxcl1, Nos2* performed (N=3, mean ± SD, **P<0.01, one-way ANOVA with Bonferroni correction). (**B**) Monocytes from aged Plt-β2M^-/-^ mice had increased reparative associated gene expression compared to WT mice. Peripheral blood monocytes were isolated and qRT-PCR for *Il10, Il27, Cxcl12* performed (N=3, mean ± SD, **P<0.01, one-way ANOVA with Bonferroni correction).

Isolated circulating monocytes from WT and Plt-β2M^-/-^ young and old mice were further characterized using quantitative real-time polymerase chain reaction (qRT-PCR) for markers of inflammatory versus reparative monocytes (Supplementary Figure 3, Supplementary Table 1). Old WT mice had a significant increase in *Cxcl1* and an insignificant, but trending, increase in *Fcgr1* and *Nos2* compared to WT young control mice (Figure 2A). Old Plt-β2M^-/-^ mice did not have a significant increase in inflammatory markers (Figure 2A). Conversely, old Plt-β2M^-/-^ mice had a significant increase in pro-reparative markers including *Il10, Il27*, and *Cxcl12* [29] compared to young genotype control mice (Figure 2B). While old WT mice had an increase in *Il10* and *Cxcl12* compared to their young genotype controls, old Plt-β2M^-/-^ had significantly more *Il10* and *Il27* compared to old WT mice (Figure 2B). The qRT-PCR data mirrored the changes in plasma levels (Figure 1E). These data suggest that a lack of platelet derived β2M blunts monocyte inflammatory differentiation and increases monocyte pro-reparative differentiation with age.

### Platelet derived β2M contributes to cardiac macrophage composition with age

**Figure 3:**
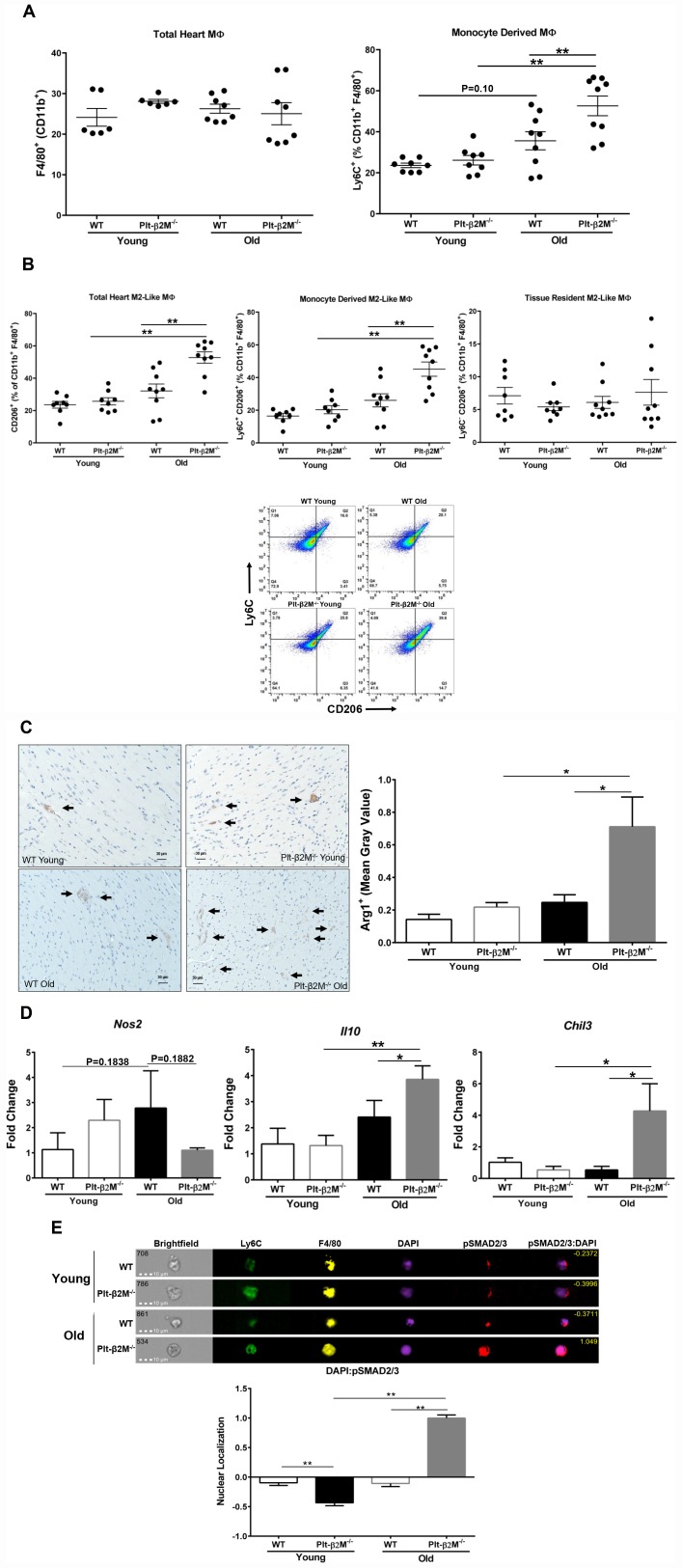
**Cardiac macrophages had different inflammatory phenotypes in older WT and Plt-β2M^-/-^ mice.** (**A**) WT and Plt-β2M^-/-^ mice had equal numbers of cardiac macrophages, but old Plt-β2M^-/-^ mice had more monocyte derived macrophages. WT old mice had a trend towards increase in monocyte derived macrophages compared to young genotype control. Flow cytometry of single cell suspensions isolated from hearts at 4 and 14 mos old mice (mean ± SEM, **P<0.01, one-way ANOVA with Bonferroni correction). (**B**) Hearts from Plt-β2M^-/-^ mice had more M2-like macrophages. Flow cytometry of single cell suspensions isolated from hearts at 4 and 14 mos (mean ± SEM, *P<0.05, one-way ANOVA with Bonferroni correction). Representative gating strategy is shown. (**C**) Hearts from Plt-β2M^-/-^ mice had more M2-like macrophages. Immunohistochemistry was performed for Arginase-1. Positive staining was observed by brown staining and quantified as mean gray value using ImageJ. Images were collected at 10x from 7 mice of WT young, WT old and Plt-β2M^-/-^ young and 9 mice of Plt-β2M^-/-^ old. Representative images shown at 20x, scale bar 30 μm (mean ± SEM, *P<0.05, 1-way ANOVA with Bonferroni correction). (**D**) Plt-β2M^-/-^ mice had greater M2-like macrophage marker gene expression. qRT-PCR for *Il10, Chil3, Nos2* were performed (N=3, mean ± SD, *P<0.05, **P<0.01, one-way ANOVA with Bonferroni correction). (**E**) Old Plt-β2M^-/-^ monocytes/macrophages in the heart, had increased pSMAD2/3 nuclear localization. ImageStream analysis was performed using single cell heart suspensions. Representative images of monocyte derived macrophages shown. Nuclear localization quantified for overlap of pSMAD2/3 with nuclear DAPI staining. Quantified images were pooled from 3 mice of young genotypes and 4 mice of old genotypes (mean ± SEM, **P<0.01, one-way ANOVA with Bonferroni correction).

Aging in both humans and mice results in expanded cardiac macrophages that arise from monocyte recruitment [12, 30]. Flow cytometric analysis of heart derived cells confirmed that there was an insignificant increase in Ly6C^+^ macrophages in the hearts of the old mice compared to young WT mice (Figure 3A). Plt-β2M^-/-^ mice had an increase in monocyte-derived cardiac macrophages, compared to WT controls (Figure 3A). This indicated that monocyte derived cardiac macrophages increased with age. Despite the difference in monocyte derived macrophages, there was no statistical difference in the total number of macrophages in the heart (Figure 3A). Because recruited circulating monocytes differentiate and become resident macrophages, we determined whether recruited circulating monocytes may be phenotypically different once in the heart. Flow cytometric analysis of heart derived cells demonstrated an increase in the total number of CD206^+^ M2-like macrophages in old Plt-β2M^-/-^ mice compared to young genotype controls and old WT mice (Figure 3B). There was no significant increase in tissue resident M2-like macrophages between genotypes and age (Figure 3B). There was also a significant increase in monocyte-derived M2-like macrophages in the hearts of old Plt-β2M^-/-^ mice compared to old WT mice (Figure 3B). This suggests that most M2-like macrophages in old Plt-β2M^-/-^ mice result from recruited monocytes. Histological sections of hearts from WT and Plt-β2M^-/-^ young and old mice were stained for Arg1 as a M2-like marker. Aged Plt-β2M^-/-^ mice had an increase in Arg1^+^ staining compared to Plt-β2M^-/-^ young and WT old (Figure 3C). RNA analysis of the heart using qRT-PCR also showed an increase in M2-like macrophage markers (*Chil3, Il10*) in Plt-β2M^-/-^ old mice compared to young Plt-β2M^-/-^ and aged WT mice (Figure 3D). Conversely, the M1-like macrophage marker (*Nos2*) was not increased in aged Plt-β2M^-/-^ mice compared to young controls (Figure 3D).

We previously demonstrated that both β2M and TGFβ activate signal transduction cascades downstream of the TGFβ receptor 1 and 2 (TGFβR) heterodimer [8]. Canonical TGFβR signaling promotes a M2-like macrophage phenotype [27]. Because monocytes and macrophages from old Plt-β2M^-/-^ mice adopted a more M2-like phenotype, we wanted to determine if the canonical signal transduction cascade was activated. Cells isolated from the heart were surface stained for markers of monocyte lineage (Ly6C) and the macrophage marker F4/80, intracellular stained for phosphorylated SMAD2/3 (pSMAD2/3), and DAPI used as a nuclear marker. Phosphorylated SMAD2/3 enters the nucleus to act as a transcription factor downstream of canonical signal transduction. ImageStream analysis was used to quantify pSMAD2/3 nuclear localization. WT and young Plt-β2M^-/-^ mice had limited pSMAD2/3 that was largely extra-nuclear (Figure 3E, representative images). However, old Plt-β2M^-/-^ mouse cardiac monocytes/ macrophages had pSMAD2/3 nuclear localization (Figure 3E).

### Lack of platelet derived β2M leads to a decline in age related heart function

**Figure 4:**
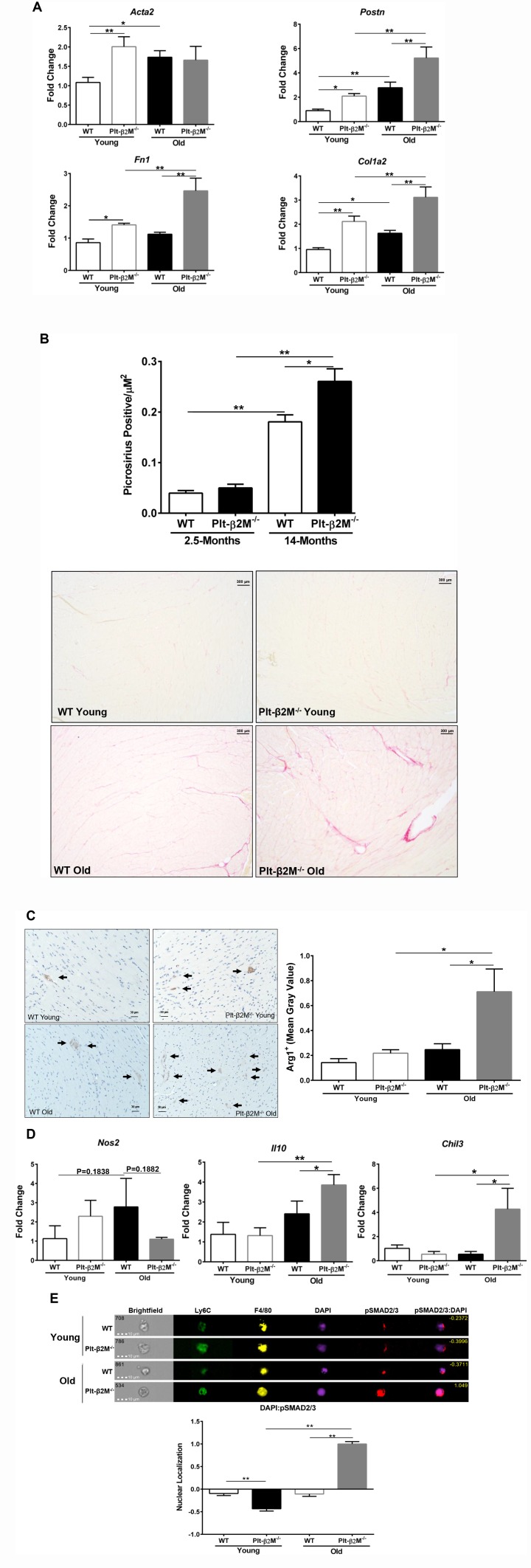
**A lack of platelet β2M increased cardiac fibrosis.** (**A**) Plt-β2M^-/-^ mice had increased activated fibroblast markers with age. RNA was isolated from single cell cardiac tissue suspensions and qRT-PCR for *Acta2, Postn, Fn1,* and *Col1a2* performed (N=3, mean ± SD, *P<0.05, **P<0.01, one-way ANOVA with Bonferroni correction). (**B**) Plt-β2M^-/-^ mice had an age associated increase in cardiac fibrosis. Representative images of Picrosirius staining of hearts from 2.5-mos and 14-mos old WT and Plt-β2M^-/-^ mice (10x magnification, scale bar=300μm). Images were pooled from 3 WT young and Plt-β2M^-/-^ young mice, 7 mice of WT old and 10 mice for Plt-β2M^-/-^ old. Image quantification was performed on ImageJ (mean ± SEM, *P<0.05, unpaired t-test with Welch’s correction). (**C**) Plt-β2M^-/-^ mice had an age-related decline in cardiac function. Echocardiography was performed on WT and Plt-β2M^-/-^ mice (mean ± SEM, *P<0.05 vs young, one-way ANOVA with Bonferroni correction). (**D**) WT and Plt-β2M^-/-^ mice had similar heart size. Whole hearts and the right tibia were isolated, heart weight determined and normalized to tibia length (mean ± SEM, one-way ANOVA with Bonferroni correction).

Recruitment of Ly6C^Lo^ pro-reparative monocytes promotes tissue fibrosis and angiogenesis, whereas Ly6C^Hi^ monocyte recruitment leads to inflammation and phagocytosis of apoptotic/necrotic cells [13, 31, 32]. Aging in both humans and mice leads to an expanded monocyte derived cardiac macrophage population [30]. The recruited cardiac macrophages promote fibroblast activation, collagen production, fibrosis, and heart dysfunction [30]. We therefore determined whether fibroblast activation and differentiation are altered with age in a platelet β2M dependent manner. Even at young ages there was an increase in transcripts for *Acta2, Fn1, Postn* and *Col1a2* in Plt-β2M^-/-^ mice compared to WT, indicative of an activated myofibroblast phenotype (Figure 4A) and old Plt-β2M^-/-^ mice had a significant increase in *Fn1, Postn,* and *Col1a2* compared to young Plt-β2M^-/-^ and old WT mice (Figure 4A). This indicates that during the aging process cardiac fibroblasts in old Plt-β2M^-/-^ mice are activated towards a myofiboblast phenotype and thus promote a pro-fibrotic environment. To measure collagen deposition, hearts from 2.5-month (young) and 14-month (old) WT and Plt-β2M^-/-^ mice were isolated, fixed, and Picrosirius stained [33]. There was no difference in collagen deposition between young WT and young Plt-β2M^-/-^ mice (Figure 4B). By 14-months old WT and Plt-β2M^-/-^ mice had more collagen deposition than their young genotype controls, however Plt-β2M^-/-^ mice had significantly more collagen than old WT mice (Figure 4B). These data indicate that during aging, fibroblasts in the hearts of Plt-β2M^-/-^ mice are activated, promoting a pro-fibrotic environment and enhanced collagen production and deposition in the heart. To determine whether there is a difference in heart function, ejection fraction (EF) and fractional shortening (FS) were measured in WT and Plt-β2M^-/-^ mice at 2.5 (young) and 13-months (old) of age by echocardiography. Young and old WT mice had no significant difference in EF and FS (Figure 4C). However, there was a significant decrease in EF and FS in old Plt-β2M^-/-^ mice compared to young genotype controls (Figure 4C), with no change in heart size (Figure 4D).

Together, these data demonstrate an important role for platelet derived β2M in age related immune homeostasis. In the absence of platelet β2M, reparative and pro-fibrotic monocyte differentiation results in age-related cardiac fibrosis. This further demonstrates the important role for platelets in normal immune development and differentiation.

## DISCUSSION

Our data shows that platelets regulate age associated monocyte immune differentiation, in a β2M dependent manner. During “inflammaging” both humans and mice adopt a more pro-inflammatory monocyte phenotype. These *in vivo* data indicate that platelets are a major source of age associated increases in plasma β2M, and that β2M is in part responsible for increased circulating Ly6C^Hi^ pro-inflammatory monocytes. We found that not only does a loss of platelet derived β2M decrease circulating Ly6C^Hi^ monocytes, but it also functionally changes monocytes by downregulating inflammatory, and upregulating reparative cytokines.

Intuitively, a decrease in Ly6C^Hi^ pro-inflammatory monocytes would be expected to be beneficial during the aging process, however, we found the opposite. There was an accelerated aging phenotype in aged Plt-β2M^-/-^ mice characterized by increased M2-macrophage markers (Figure 3B–3D), increased myofibroblast activation markers (Figure 3E), increased collagen deposition (Figure 4A–4B), and decreased heart function (Figure 4C). These data indicate that a lack of inflammatory monocytes may not be cardiac protective if accompanied by a shift to more pro-reparative monocytes that leads to excess fibrosis. Inflammation is clearly bad in some contexts, but these data suggest that it may also be critical at some level for maintaining heart function through the aging process. This also highlights the concept that inflammation in general may not be bad or good, but rather depends on the time, duration, and disease context. Therefore, what may be more important than strictly preventing inflammation is maintaining an appropriate immune homeostasis.

Even in healthy humans, the natural process of aging leads to dynamic changes to heart anatomy and physiology [34]. Multiple previous experiments analyzing the collagen content of young and elderly human hearts showed that there was an increase in amount of collagen, collagen fiber diameter and a shift towards type I fibers during aging in humans, leading to increased interstitial fibrosis [35–37]. We have shown in this study and our previous study, that lack of platelet derived β2M, enhances TGFβ signal transduction [8]. TGFβ signaling is a potent inducer of collagen production by transdifferentiated fibroblast [38] and has long been hypothesized as a major contributor to age-related collagen deposition and fibrosis in humans [35]. A major hurdle in treating humans with cardiac fibrosis is that we lack effective pharmacological therapies [39]. Our study may help in understanding the pathogenesis of the aging heart dysfunction and therefore contribute to future treatment strategies.

While it is clear that β2M is not the only platelet derived protein that can influence monocyte polarization *in vivo*, these data, and our past studies, demonstrate that β2M does have a major role in maintaining monocyte phenotypes in basal conditions as well as in a disease context. This study is a clear distinction from our previous work on platelet-derived β2M because we previously reported the ability to regulate monocyte polarization, macrophage phenotype, and cardiac function after a pathological insult to the heart using a myocardial infarction (MI) ligation of the left anterior descending artery model. This study implicates platelet-derived β2M as a systemic aging factor that influences monocyte phenotype, macrophage composition, and cardiac output, *in vivo* during the natural aging phenomenon without an additional pathological insult.

A recent study has shown that in the heart cardiomyocytes are a major source of β2M and it has a role in fibroblast activation in a transverse aortic constriction (TAC) model, opening the door for more exciting research on non-MHC I trafficking roles for β2M [40]. In our study we cannot rule out a platelet – CD8^+^ T cell interaction component to the phenotype in the Plt-β2M^-/-^ mice. Although we saw no changes in total T cell or CD8^+^ numbers, our mice are only aged to 14-16 months which is more like middle-aged humans (40-60 years) than truly elderly patients [41]. Human data suggests that the total number of T cells, and specifically CD8+ T cells, doesn’t change between young (<40 years) and middle-aged (40-60 years) groups, however it significantly decreases in the old (>60 years) age group [42]. Perhaps if mice were aged >18-months we may see a T cell dependent effect. Future studies will be needed to study the effects of monocyte subsets on the aging process and how monocytes, macrophages and fibroblast cross-talk to regulate the aging heart function.

## MATERIALS AND METHODS

### Reagents

Anti-mouse APC MHC Class I/H-2Db (17-5958-82/AF6-88.5.5.3), mouse APC CD4 (17-0041-82/ OX35) were purchased from eBioscience. Flow cytometry antibodies to anti-mouse/human APC CD11b (101212/M1/70), anti-mouse PerCP/Cy5.5 CD115 (347310/AFS98), mouse FITC Ly6C (128006/HK1.4), mouse PE CD182/CXCR2 (149303/SA044G4), mouse PerCP/Cy5.5 CD184/CXCR4 (146510/L276F12), mouse FITC CD3 (100306/145-2C11), mouse PE F4/80 (123146/BM8), mouse PerCP-Cy5.5 CD206 (141715/ C068C2) were purchased from BioLegend. Antibody to anti-mouse FITC Ly6G (551460/1A8), mouse PE CD8a (553033/53-6.7), and mouse Alex Fluor 647 Smad2 (pS465/pS467)/Smad3 (pS423/pS425) (562696/O72-670) were purchased from BD Biosciences. ImageStream antibody to nuclear stain 4',6-Diamidino-2-Phenylindole, Dihydrochloride (DAPI, D1306) was purchased from Thermo Fisher Scientific.

ELISA kit for mouse β2M (LS-F14141) was purchased from LifeSpan Bioscience. Mouse TGF-beta 1 (MB100B) quantikine ELISA was purchased from R&D Systems.

### Mouse studies

All mice used in these experiments were on a C57BL6/J background. Both male and female mice were used in the experiments as we have previously found no difference between genders in our mouse model [8]. The generation of the PF4^Cre^-β2M^Flox/Flox^ mice has been previously described [8]. To define a “young” age group, all mice were under 4 months of age at the time of harvest. To define the “old” age group, mice were 13 months or older. We acknowledge that our “old” age group does correlate with more “middle aged” in humans according to Jackson Laboratory, however an accelerated aging phenotype was observed.

Mice were bled via retro-orbital route into EDTA coated capillary blood collection tubes (Greiner Bio-One). Complete blood counts (CBCs) of the whole blood were measured using Abaxis VetScan HM5. Plasma was isolated from whole blood collected in EDTA by spinning at 800 rcf (3000 rpm) for 10 minutes and collecting the top layer. Diluted blood was stained for flow cytometry, then fixed with BD FACS Lysing Solution (BD Biosciences, 349202). Monocytes were analyzed by flow cytometry first gating on double positive CD11b and CD115 cells; from there monocytes that were high in Ly6C expression were gated and quantified. Neutrophils were gated on by double positive population of CD11b and Ly6G then subsets were subdivided into populations that were high for CXCR2 or CXCR4. T cells were identified by gating on positive CD3 cells then further subdivided and represented as a percentage of cells also positive for either CD4 or CD8.

At the time of harvest mouse hearts were weighed, minced and placed in digestion buffer at 37°C for 1 hr while rotating. Right tibias of mice were collected and measured to normalize heart weight. The digestion buffer contained Dulbecco's Modified Eagle Medium (Gibco, 10566016), 1 mg/mL collagenase type II (Worthington, LS004176), 2.5% fetal bovine serum (Thermo Fisher Scientific, 10437028), 1 mM HEPES (Corning, 25060CL), 1mM EDTA (Invitrogen, 15575020). The digested hearts were passed through a 100 μm mesh nylon strainer (Corning, 352360). Any residual red blood cells (RBCs) were lysed with ACK lysis solution (Gibco, A1049201). Single cell isolates were centrifuged at 300 rcf (1250 rpm) for 5 minutes. The resulting cell pellet was either resuspended into RLT buffer (Qiagen) for qRT-PCR or 1x PBS for flow cytometry or ImageStream. Total macrophages in the heart were identified by flow cytometry and gated on double positive CD11b and F4/80 cells. Monocyte derived macrophages were quantified and represented by gating on cells positive for Ly6C of double positive CD11b, F4/80. Total heart M2-like macrophages were gated as percentage CD206 positive from the CD11b, F4/80 double positive population. Monocyte-derived and tissue resident M2-like macrophages were characterized as positive for CD11b, F4/80, CD206 and either positive for Ly6C or negative, respectively.

Primary monocytes were isolated from whole blood of mice. Blood from mice retro-orbitally bled into EDTA was treated with ACK lysis solution to remove RBCs. The blood was then spun down at 300 rcf (1250 RPM) and the cell pellet was resuspended into isolation buffer. Isolation buffer was made up of 1X PBS (Fisher BioReagents, BP39920), 1 mM EDTA, and 2.5% FBS. Monocytes were isolated from the resuspended cell solution using an EasySep^TM^ Mouse Monocyte Isolation Kit (STEMCELL Technologies, 19861) according to manufacturer’s instructions. Isolated monocytes were resuspended into RLT lysis buffer for qRT-PCR analysis.

### Quantitative real-time polymerase chain reaction

Purified monocytes and single cell isolated heart tissue resuspended in RLT lysis buffer were used to extract RNA using RNeasy Mini Kit (Qiagen, 74106). The concentration of RNA was measured using NanoDrop^TM^ 2000 (Thermo Fisher Scientific). Isolated RNA was made into cDNA using High Capacity RNA-to-cDNA Kit (Applied Biosystems, 4387406). Gene expression was measured through qRT-PCR using TaqMan® gene expression master mix (Thermo Fisher Scientific, 4369016) protocol on the BioRad iCycler iQ5 (1708740).

Taqman primers for quantitative real-time polymerase chain reaction (qRT-PCR) of Fcgr1 (Mm00438874_m1), Cxcl1 (Mm04207460_m1), Il10 (Mm01288386_m1), Il27 (Mm00461162_m1), Arg1 (Mm00475988_m1), Cxcl12 (Mm00445553_m1), Chil3 (Mm00657889_mH), Nos2 (Mm00657889_mH), Acta2 (Mm00725412_s1), Postn (Mm01284919_m1), Fn1 (Mm01256744_m1), Col1a2 (Mm00483888_m1) were purchased from Thermo Fisher Scientific.

### Immunohistochemistry

Mouse hearts were collected and placed into fixative solution (10% neutral buffered formalin). Hearts were cross sectioned, paraffin embedded and cut into 5 μM sections. Collagen fibers were histologically visualized using Picro Sirius Red Stain Kit (Abcam, ab150681) according to the manufacturer’s instructions. M2-macrophages were visualized using Arginase-1 antibody (Cell Signaling Technology, 93668S).

For immunostaining, slides were deparaffinized and rehydrated and placed into 3% H_2_O_2_ for 15 minutes. Slides were washed with TBS 3 times. In a pressure cooker slides were incubated in Dako Target Retrieval Solution (S1699) for 15 minutes, washed in PBS, and then incubated in Dako Protein Block (X0909) for 30 minutes. Anti-Arg1 was diluted 1:500 and incubated overnight at 4°C. Slides were rinsed in PBS and incubated in biotinylated anti-rabbit antibody (Vector Laboratories, BA-1000, 1:250 in Dako Antibody Diluent) for 30 minutes at room temperature. Slides were rinsed and incubated with VECTASTAIN® Elite® ABC-HRP Kit (Vector Laboratories, PK-6100) for 30 minutes. Slides were rinsed in PBS again and DAB Peroxidase (HRP) Substrate (Vector Laboratories, SK-4100) was added for 5 minutes. Slides were washed in dH_2_0 for 5 minutes, counterstained, and coverslip added. As a negative control, rat IgG2b was used in the primary antibody step.

Histological images were imaged at 10x (quantification) and 20x (representative images) magnification, using a BX41 microscope with SPOT camera and SPOT Basic imaging software. Arg1 staining was quantified using ImageJ. Color deconvolution 1.5 plug-in H DAB vector [43]. Colour 2 (DAB) channel was used to visualize brown positive stained Arg1+ cells. The threshold was adjusted to upper slider 0, lower slider 210 for all images for consistency to distinguish between positive cells and negative.

Picrosirius staining was quantified using ImageJ. Each image was converted to grayscale using the RGB Stack command. Under the green channel, the threshold was adjusted to upper slider 0, lower slider 210 for all images for consistency to distinguish between collagen (red) and tissue (yellow). The resulting image was quantified for pixel intensity and normalized to tissue surface area.

### Data analysis

All flow cytometry was run on either an Accuri C6 or BD LSR II. FlowJo version 7.6 was used to analyze FACS samples. ELISAs were analyzed using a four-parameter logistic (4-PL) curve-fit. Gene expression of qRT-PCR was analyzed in Microsoft Excel using calculations for fold change 2^(-delta delta CT)^ with GAPDH as gene of reference and normalized to WT young. All experiments are representative and were repeated at least twice.

### Statistical analysis

Statistical tests were performed using GraphPad Prism. When two independent groups were compared a student t-test, 2-tailed, was used. For experiments containing more than two independent groups one-way ANOVA with Bonferroni correction was used. All statistical tests with a P-value of <0.05 were considered statistically significant and graphically represented by 1 star. Any P-value <0.01 were graphically represented by 2 stars. All data is represented as mean ± standard error of the mean (SEM) or standard deviation (SD) based on figure legend.

### Study approval

All mouse work conducted in this study was approved by the University of Rochester Institutional Animal Care and Use Committee under protocol number 2009-022.

## Supplementary Material

Supplementary Figures

Supplementary Tables
