# Supplementary material for: Platelet-derived β2m regulates age related monocyte/macrophage functions
Source: Aging (Albany NY). 2019 Dec 18;11(24):11955–74. doi: 10.18632/aging.102520 (PMC6949047; doi:10.18632/aging.102520)
Supplement: Supplementary Figures [file aging-11-102520-s001..pdf]

SUPPLEMENTARY FIGURES

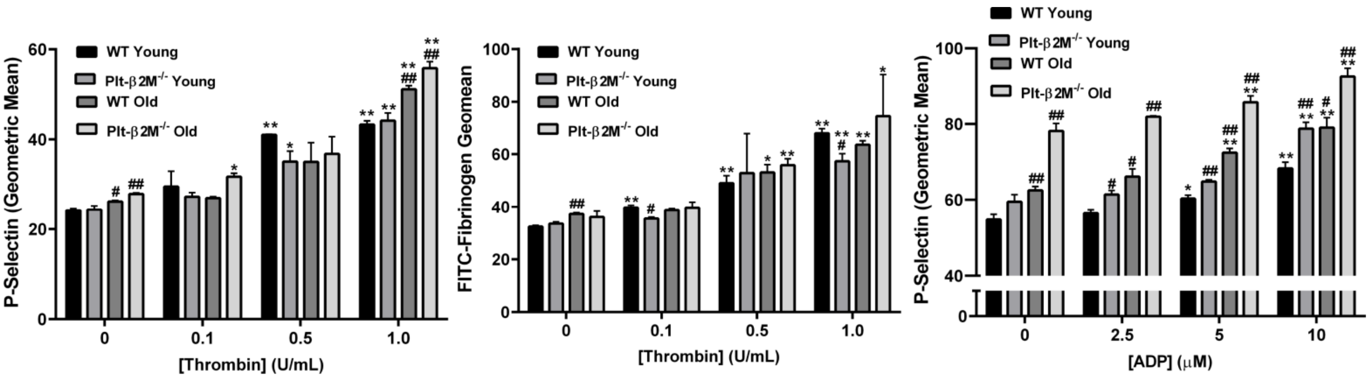

Supplementary Figure 1. Platelets from old WT and old Plt-β2M$^{-/-}$ are hyper-activatable. Washed WT and Plt-β2M$^{-/-}$ platelets were stimulated with thrombin or ADP and surface P-selectin was measured by flow cytometry. Platelets were also thrombin stimulated and FITC tagged fibrinogen binding determined by flow cytometry (N=4, *P<0.05 vs 0, **P<0.01 vs 0, #P<0.05 vs WT young, ##P<0.01 vs WT young, mean ± SEM, multiple t tests using Holm-Sidak multiple comparison correction).

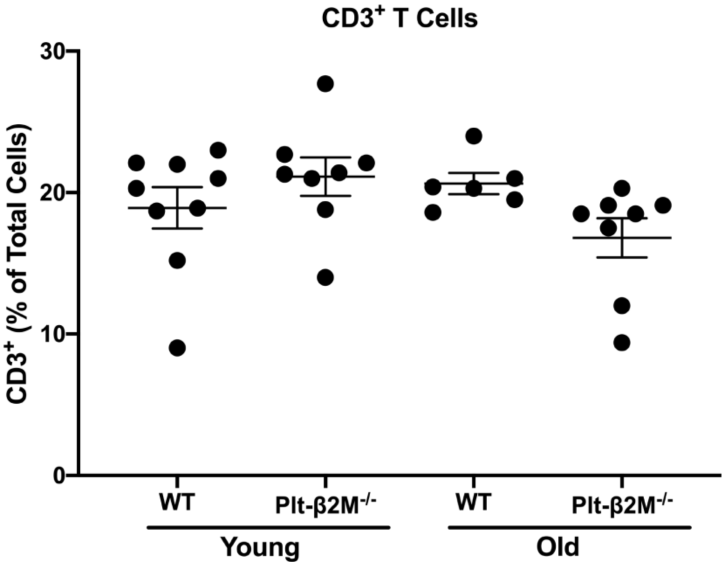

Supplementary Figure 2. WT and Plt-β2M$^{-/-}$mice had similar numbers of circulating CD3^{+} cells throughout aging. Peripheral blood was isolated from 4 mos and 14-16 mos old WT and Plt-β2M$^{-/-}$mice and CD3 cells were quantified by flow cytometry (N=6-8, mean ± SEM, one-way ANOVA with Bonferroni correction).

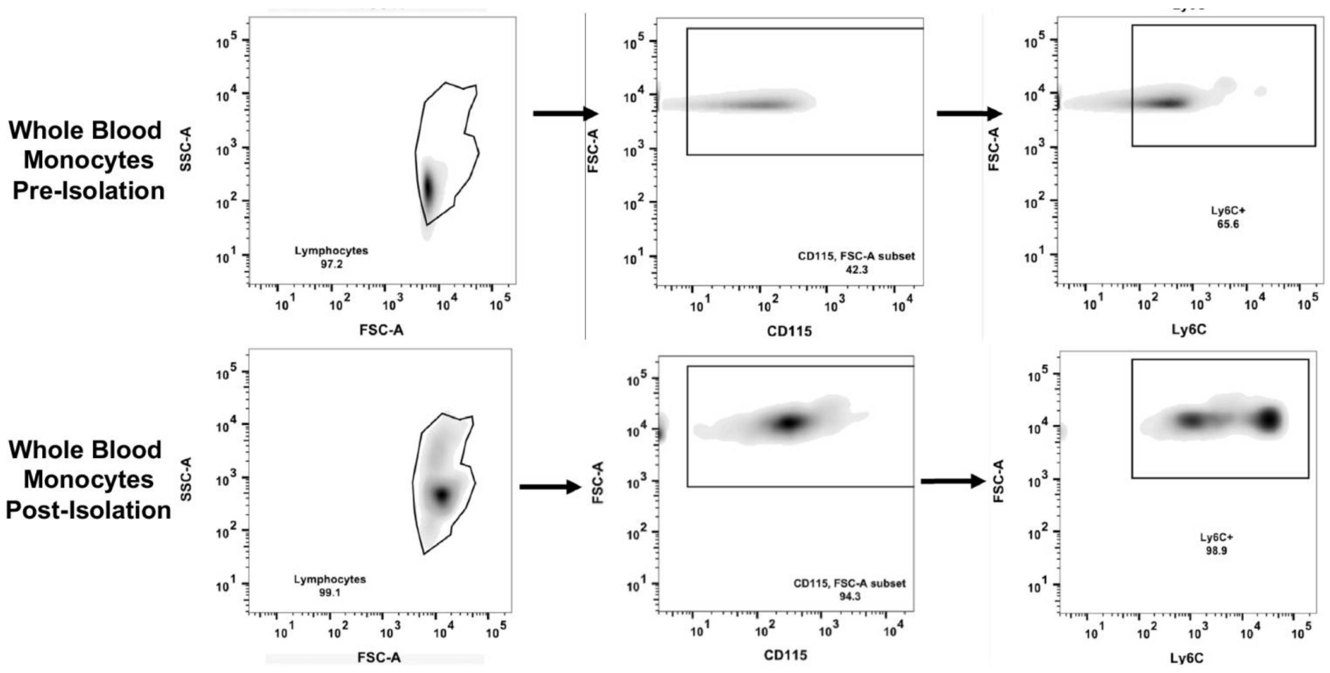

Supplementary Figure 3. Monocytes isolated from negative selection kit for qRT-PCR about 95% pure. Monocytes were stained for surface expression of CD115, Ly6C and measured by flow cytometry. Top panel: cells from the whole blood. Bottom panel: cells from whole blood after negative selection.
