# Supplementary material for: Platelet-derived β2m regulates age related monocyte/macrophage functions
Source: Aging (Albany NY). 2019 Dec 18;11(24):11955–74. doi: 10.18632/aging.102520 (PMC6949047; doi:10.18632/aging.102520)
Supplement: Supplementary Tables [file aging-11-102520-s002..pdf]

SUPPLEMENTARY TABLES

Supplementary Table 1. RNA isolated from the heart and whole blood monocytes lack contaminants. RNA concentrations and absorbance measured by nanodrop. Concentration of nucleic acid and Absorbance of 260/280 are represented as average ± SEM. A ratio of A260/A280 of ~2.00 is generally accepted as "pure" for RNA.

	Heart		Isolated Monocytes	
**Mouse**	**[Nucleic Acid] (ng/µL)**	**A260/A280 (Abs)**	**[Nucleic Acid] (ng/µL)**	**A260/A280 (Abs)**
**WT Young**	65.38 ± 18.35	2.06 ± 0.02	7.80 ± 2.36	2.55 ± 0.22
**Plt-β2M$^{-/-}$ Young**	38.08 ± 9.06	2.01 ± 0.02	8.88 ± 2.11	2.21 ± 0.14
**WT Old**	57.72 ± 20.97	2.02 ± 0.03	21.57 ± 12.03	2.05 ± 0.11
**Plt-β2M$^{-/-}$ Old**	36.50 ± 13.84	2.08 ± 0.04	16.40 ± 3.63	2.09 ± 0.02
